# Probing Amphotericin B Single Channel Activity by Membrane Dipole Modifiers

**DOI:** 10.1371/journal.pone.0030261

**Published:** 2012-01-19

**Authors:** Olga S. Ostroumova, Svetlana S. Efimova, Ludmila V. Schagina

**Affiliations:** Institute of Cytology of the Russian Academy of Sciences, St. Petersburg, Russia; National Cancer Institute, United States of America

## Abstract

The effects of dipole modifiers and their structural analogs on the single channel activity of amphotericin B in sterol-containing planar phosphocholine membranes are studied. It is shown that the addition of phloretin in solutions bathing membranes containing cholesterol or ergosterol decreases the conductance of single amphotericin B channels. Quercetin decreases the channel conductance in cholesterol-containing bilayers while it does not affect the channel conductance in ergosterol-containing membranes. It is demonstrated that the insertion of styryl dyes, such as RH 421, RH 237 or RH 160, in bilayers with either cholesterol or ergosterol leads to the increase of the current amplitude of amphotericin B pores. Introduction of 5α-androstan-3β-ol into a membrane-forming solution increases the amphotericin B channel conductance in a concentration-dependent manner. All the effects are likely to be attributed to the influence of the membrane dipole potential on the conductance of single amphotericin B channels. However, specific interactions of some dipole modifiers with polyene-sterol complexes might also contribute to the activity of single amphotericin B pores. It has been shown that the channel dwell time increases with increasing sterol concentration, and it is higher for cholesterol-containing membranes than for bilayers including ergosterol, 6-ketocholestanol, 7-ketocholestanol or 5α-androstan-3β-ol. These findings suggest that the processes of association/dissociation of channel forming molecules depend on the membrane fluidity.

## Introduction

The structure of polyene antibiotic amphotericin B (AmB) comprises two rigid fragments, a macrolide ring and a mycosamine sugar moiety, which are linked by a β-glycosidic bond. The so-called “polar head” of the molecule contains carboxyl and amino groups. For over forty years, the AmB has been one of the most important agents used to combat systemic fungal infections. In spite of side effects such as nephrotoxicity, anemia, and cardiac arrhythmia [1]–[2], AmB remains the drug of choice for treatment of immunosuppressed patients, such as cancer patients in intensive chemotherapy, solid organ transplant recipients, and AIDS patients. Pharmaceutical technologies provide innovative formulations, which aim to reduce the concentration of the free AmB in the patient serum without harming its therapeutic efficacy.

Sensitive target organisms lose their cellular integrity due to AmB-induced pore formation in their membranes. The exact molecular architecture of the AmB channel is under debate; different models for the formation and structure of the AmB channel have been proposed. The most popular is the sterol-dependent double-pore model: the two-sided effect of polyene antibiotic results from the association of AmB with sterol molecules and the formation of anion-selective symmetric barrel stave pores made from two “half-pores” in opposite monolayers [3]–[6].

Sterol-dependent membrane activity of AmB suggests that the observed therapeutic efficacy of AmB might be related to a differential preference between sterols found in cell membranes. In mammalian cells, cholesterol (Chol) is the major membrane sterol, whereas in fungi it is ergosterol (Erg) [7]. It is not still clear whether the therapeutic effect of AmB is caused by the preferential formation and stability of a complex of polyene and ergosterol over cholesterol [8]–[11] or the observed effects result from the different influences of both sterols on structural and dynamical properties of the membrane [12]–[14].

Sterols are responsible for the membrane fluidity. The main feature of the phosphatidylcholine∶cholesterol membrane phase diagram is the presence of an ordered phase at bilayer concentrations of more than 25 mol% cholesterol [15]. Biological membranes contain substantial amounts of cholesterol or equivalent sterols and the phase segregation is expected for many biological membranes. AmB molecules exhibit higher affinity toward the sterol-containing lipid-ordered phase (rafts) and, therefore, might be cumulated in rafts [14]. AmB causes an increase in the internal order of membranes formed with saturated lipids and Chol, while AmB brings about fluidization in the centre of the bilayers with the same amount of Erg [14], [16], [17].

Czub and Baginski [14] showed that in a membrane, the negatively charged carboxyl group (COO^−^) of AmB is shifted slightly toward the aqueous phase as compared to the protonated amino group (NH_3_^+^). The authors suggested that the AmB head dipoles may influence on the membrane dipole potential (ϕ*_d_*) drop. The dipole potential of the membrane originates from the specific orientation of dipole moments of the lipid molecules and the adjacent water dipoles in the interfacial region. Depending on the structure of lipids, its magnitude can vary from 100 to 400 mV, with positive values in the membrane interior [18]–[20]. It is known that sterols modulate the properties of a bilayer not only in its fluidity but also in the membrane dipole potential [21]. Thus, if the membrane dipole potential can ensure a significant contribution to the regulation of AmB channel activity, membrane dipole modifiers might be useful for chemotherapeutical investigations to design less toxic preparations with enhanced therapeutic effectiveness.

The present study is an attempt to examine the effects of dipole modifiers and their structural analogs including various sterols, flavonoids, and styryl dyes on the single AmB channel properties. The roles of the membrane dipole potential, membrane fluidity and specific interactions between dipole modifiers and polyene-sterol complexes in the single channel activity of AmB are discussed.

## Materials and Methods

All chemicals were of reagent grade. Synthetic 1,2-diphytanoyl-*sn*-glycero-3-phosphocholine (PC), cholesterol (Chol), ergosterol (Erg), and 5α-androstan-3β-ol were obtained from Avanti Polar Lipids, Inc. (Pelham, AL). Phloretin (3-(4-hydroxyphenyl)-1-(2,4,6-trihydroxyphenyl)-1-propanone), phloridzin (1-[2-(β-D-Glucopyranosyloxy)-4,6-dihydroxyphenyl]-3-(4-hydroxyphenyl)-1-propanone), genistein (5,7-Dihydroxy-3-(4-hydroxyphenyl)-4H-1-benzopyran-4-one), genistin (Genistein-7-O-β-D-glucopyranoside), 2′,4′,6′-trihydroxy-acetophenone monohydrate, quercetin (2-(3,4-Dihydroxyphenyl)-3,5,7-trihydroxy-4H-1-benzopyran-4-one), 6-ketocholestanol, 7-ketocholestanol (5-cholesten-3β-ol-7-one) were purchased from Sigma Chemical (St. Louis, MO), RH 421 (N-(4-sulfobutyl)-4-(4-(4-(dipentylamino)phenyl)butadienyl) pyridinium, inner salt), RH 237 (*N*-(4-sulfobutyl)-4-(6-(4-(dibutylamino)phenyl)hexatrienyl)pyridinium, inner salt), and RH 160 (*N*-(4-sulfobutyl)-4-(4-(4-(dibutylamino)phenyl)butadienyl)pyridinium, inner salt) from Molecular Probes (Eugene, OR). Water was distilled twice and deionized. 2 M KCl solutions were buffered with 5 mM Hepes, pH 7.0. Amphotericin B from *Streptomyces* sp. (AmB) was purchased from Sigma Chemical (St. Louis, MO).

Virtually solvent-free planar lipid bilayers were prepared according to a monolayer-opposition technique [22] on a 50-µm-diameter aperture in the 10-µm thick Teflon film separating two (*cis* and *trans*) compartments of the Teflon chamber. The aperture was pretreated with hexadecane. Lipid bilayers were made from PC and sterol (Chol, Erg, 5α-androstan-3β-ol, 6-ketocholestanol, or 7-ketocholestanol) in different molar ratios. After the membrane was completely formed, AmB from a stock solution (0.1 mg/ml DMSO) was added to both compartments to obtain a final concentration that ranged from 10^−8^ to 10^−6^ M. Ag/AgCl electrodes with agarose/2 M KCl bridges were used to apply the transmembrane voltage (*V*) and measure the transmembrane current. “Positive voltage” refers to the case in which the *cis*-side compartment is positive with respect to the *trans*-side. All experiments were performed at room temperature. Final concentration of DMSO in the chamber did not exceed 10^−4^ mg/ml.

The two-side addition of phloretin, phloridzin, genistein, genistin, 2′,4′,6′-trihydroxy-acetophenone, quercetin, RH 421, RH 237, or RH 160 from stock mM solutions in ethanol or DMSO to the membrane-bathing solution yielding final concentrations of 20 µM for different flavonoids and 5 µM for various RH dyes was used to modulate AmB activity. Noticed concentrations of ethanol and DMSO in the bilayer bathing solutions did not affect membrane properties (resistance, capacity and stability).

Current measurements were carried out using an Axopatch 200B amplifier (Axon Instruments) in the voltage clamp mode. Data were digitized by Digidata 1440A and analyzed using pClamp 10 (Axon Instruments) and Origin 7.0 (Origin Lab). Current tracks were filtered by 8-pole Bessel 100 kHz. The total number of events, *N*, used for the plotting the histograms of transmembrane current fluctuations at a fixed value of transmembrane voltage ranged from 300 to 8000. The histograms were approximated by the functions of normal distribution. The channel conductance, *G*(*V*), was determined as the ratio of central value of the current, *I*, to transmembrane voltage, *V*. The lifetime of channels was determined only in the cases of one current level i.e. functioning of one single channel. The total number of measurements used for histogram construction ranged from 150 to 7000. The value of mean channel lifetime, *τ*, was defined as a parameter of the exponential function approximating the obtained distribution. The distribution hypothesis was verified using χ^2^ (P<0.05).

Since a sterol concentration and a phase separation in the membrane (the presence of lipid rafts, which are able to cumulate sterols and AmB), may affect the parameters of AmB-channels, we compared the conductance-voltage characteristics of single pores at different concentrations of cholesterol or ergosterol in the membrane forming solutions: 5 mol%, 33 mol% and 67 mol%. It was found that the conductance-voltage characteristics are the same for these cases. Therefore, for experiments with dipole modifiers, a 33 mol% sterol concentration was chosen, because it is close to the amount of sterols in biological membranes and thus allows to simulate the cellular situation.

Changes in K^+^-nonactin steady-state conductance were measured to estimate the changes of the membrane dipole potential after the addition of quercetin or methyl-β-cyclodextrin into a bilayer bathing solution (0.1 M KCl, 5 mM Hepes, 7.4). The corresponding calculations were performed assuming that the membrane conductance is related to the bilayer dipole potential by the Boltzmann distribution [23]: 
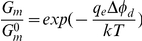
, where *G_m_* and *G_m_^0^* are the steady-state membrane conductance induced by K^+^-nonactin in the presence and in the absence of quercetin or methyl-β-cyclodextrin, respectively, *Δϕ_d_* are the changes of the membrane dipole potential after the addition of quercetin or methyl-β-cyclodextrin into a bilayer bathing solution; *q_e_*, *k*, *T* have their usual meanings.

## Results and Discussion

**Figure 1:**
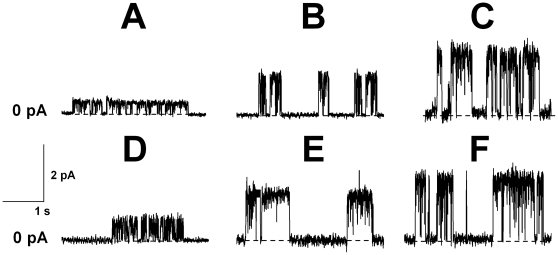
Current fluctuations corresponding opening and closing of the single AmB-channels in the planar lipid bilayers. The membranes were made from the PC∶Chol (67∶33 mol%) and bathed in 2 M KCl 5 mM Hepes pH 7.0. *V* = 200 mV. Bilayer bathing solutions contain: (**A**) – 20 µM phloretin, (**B**) – no dipole modifiers, (**C**) – 5 µM RH 421, (**D**) – 20 µM quercetin, (**E**) – 5 µkM RH 160, (**F**) – 5 µM RH 237.

Figure 1 illustrates the effect of different membrane dipole modifiers on single AmB-channels in lipid bilayers formed from PC∶Chol (67∶33 mol. %) and bathed in a 2 M KCl pH 7.0 solution. Upper panel of Fig. 1 presents current fluctuations in the presence of 20 µM phloretin (A), no dipole modifiers (B), and 5 µM RH 421 (C). The addition to the membrane bathing solution of phloretin, which is known to decrease the membrane dipole potential [23], [24], produced a significant decrease of the channel conductance (by factor of 3), while the pore conductance increased by ∼1.5 in the presence of RH 421, known to increase ϕ*_d_* [25]. For preferentially anion-conductive AmB-channels [26] one could expect that a decrease of ϕ*_d_* would produce a decrease of the pore conductance [23], [27]. We have previously observed similar effects of these dipole modifiers on predominantly anion-selective syringomycin E channels [28]. The opposite effect (increase of the channel conductance with decreasing ϕ*_d_*) was observed for cation-selective channels produced by gramicidin A, alamethicin, and surfactin [29]–[33].

We applied different analogs of phloretin, phloridzin, genistein, genistin, 2′,4′,6′-trihydroxy-acetophenone monohydrate, and quercetin. It was observed that phloridzin does not affect the AmB-channel conductance (data not shown). This finding is in agreement with the fact that phloridzin is several orders of magnitude less effective on the K^+^-nonactin conductance of lecithin∶cholesterol (20∶80 mol%) membranes than phloretin [23]. Compounds which have the exiguous effect on the membrane dipole potential, genistein, genistin, and 2′,4′,6′-trihydroxy-acetophenone [30], [34], did not practically influence the AmB-channel amplitude (data not shown). The addition of up to 20 µM of quercetin to the membrane bathing solution led to a significant reduction of AmB-pore current amplitude (Fig. 1D). One can assume that the introduction of quercetin leads to some reduction of ϕ*_d_* similar to phloretin. Indeed, we found that the addition of quercetin in the solution bathing PC∶Chol-membrane led to significant increase of K^+^-nonactin steady-state conductance. Increase in the cation conductance means a reduction of the membrane dipole potential. Introduction of 20 µM quercetin corresponds to ϕ*_d_* reduction on 100±10 mV (Δϕ_d_ = −100±10 mV) (see Materials and methods).

We also used analogs of RH 421, RH 237 and RH 160. Malkov and Sokolov [35] have shown that among these dyes RH 421 has the strongest effect on increasing dipole potential of PC-membranes. RH 237 has an intermediate effect and RH 160 has the smallest. The observed increase of the AmB-pore conductance correlates with the dipole potential changes induced by these RH molecules (compare Fig. 1C, F, E).

Thus, the obtained results show that the membrane dipole potential reduction is followed by decreasing AmB channel conductance. It should be noted that Asandei and Luchian [36] attributed the pH-induced changes of the single-molecule ionic conductance of AmB-channels to variations of the dipole membrane potential.

**Figure 2:**
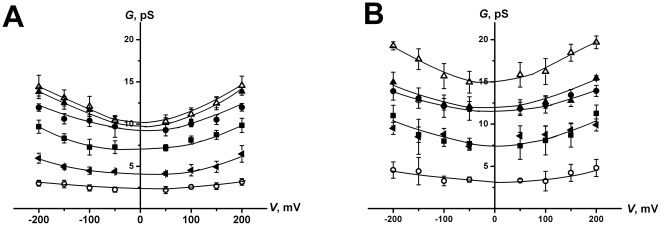
Conductance-voltage curves of the single AmB-channels. The membranes were made from PC∶Chol (67∶33 mol%) (**A**) and PC∶Erg (67∶33 mol%) (**B**), and bathed in 2.0 M KCl 5 mM Hepes pH 7.0. Bilayer bathing solutions contain: (▪) – no dipole modifiers, (○) – 20 µM phloretin, (◂) – 20 µM quercetin, (▵) – 5 µM RH 421, (▴) – 5 µM RH 237, (•) – 5 µM RH 160.

The sterol-dependent membrane activity of AmB forced us to investigate AmB-channels in bilayers containing different sterols, especially ergosterol. Figure 2 shows conductance-voltage curves in the absence and in the presence of phloretin, quercetin, and various RH dyes in the solutions bathing PC∶Chol (67∶33 mol. %) (A) and PC∶Erg (67∶33 mol%)-bilayers (B). The data show that the AmB-pore conductance is the same for Erg- and Chol-containing bilayers in the absence of any other agents (channel conductance at zero transmembrane voltage, *G^0^*≈7 pS). For Erg- and Chol-containing bilayers the effect of phloretin is also the same (*G^0^*≈2.5 pS). RH 421 is more effective in Erg-containing bilayers (in the presence of RH 421 *G^0^*≈15 pS) than in Chol-containing membranes (*G^0^*≈10 pS), while the pore conductance in the presence of RH 237 and RH 160 is practically the same for Erg- and Chol-containing bilayers. It should be noted that, as well as in Chol-containing membranes, genistein, genistin, and 2′,4′,6′-trihydroxy-acetophenone, do not affect the AmB-channel conductance in Erg-containing bilayers (data not shown). In contrast to Chol-containing bilayers, quercetin does not affect the AmB-channel conductance in Erg-containing membranes. Different effects of RH 421 and quercetin on the bilayers containing these sterols are likely to be attributed to interactions between these modifiers and amphotericin-sterol complexes. Recently, the specific interaction of 5- and 4′-hydroxylated flavonoids (for example, phloretin and genestein) with the voltage sensor of alpha-hemolysin pore was demonstrated [37].

**Figure 3:**
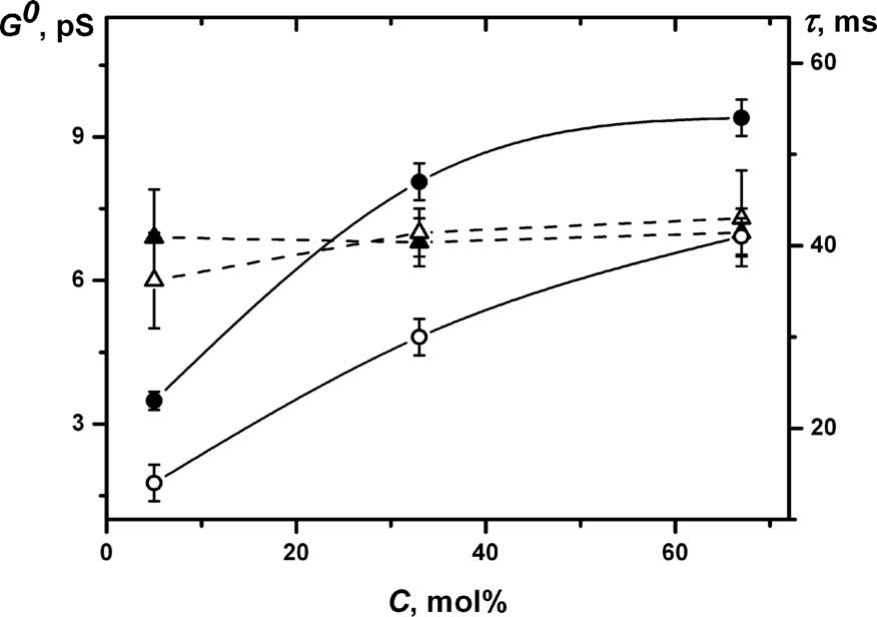
The dependences of the conductance at zero transmembrane voltage, *G^0^* (dash lines), and mean dwell time, *τ* (solid lines), of AmB-channels on the concentration of Chol (solid symbols) or Erg (open symbols) in the membrane forming PC-solution. The membranes were bathed in 2.0 M KCl 5 mM Hepes pH 7.0.

Figure 3 presents the dependences of the conductance at zero transmembrane voltage and mean dwell time of AmB-channels as functions of the Chol- or Erg-concentration in the membrane-forming solution. One can see that the pore conductance does not depend on the sterol concentration. In both cases, channel dwell time increased with increasing sterol concentration in the membranes. As the cholesterol-induced change in the membrane dipole potential is biphasic (cholesterol increases ϕ*_d_* in the concentration range from 0 to 35 mol%, a maximum was observed at 35–45 mol%, after which ϕ*_d_* starts to decrease) (see Fig. 3 in [38]), the observed monotonic increase of the channel dwell time with increasing sterol concentration can hardly be discussed in terms of membrane dipole potential changes. This fact may be rationalized in terms of membrane fluidity, assuming that an increase in the sterol concentration leads to a condensation effect in the bilayer [39], [40], which in turn hinders dissociation of two half-pores. It can also be noticed that the pore life time is higher for Chol-containing membranes than for Erg-containing bilayers. These data are also in agreement with the fact that AmB increases the internal order of bilayers containing Chol, while it has no effect on the order of the bilayer with Erg [14].

We probed the AmB-channel activity with other sterols. Table 1 presents the effect of 6-ketocholestanol, 7-ketocholestanol and 5α-androstan-3β-ol. 6-ketocholestanol and 7-ketocholestanol are known to increase and decrease ϕ*_d_*, respectively (see Fig. 3 in [38]). AmB-channel conductance *G^0^* does not depend on the 6-ketocholestanol concentration in the membrane forming solution and is equal to approximately 7 pS. In case of 67 mol% of 7-ketocholestanol *G^0^* = 4±1 pS. Since 6-ketocholestanol increases the ϕ*_d_*-value much more efficiently compared to the decrease caused by 7-ketocholestanol (see Fig. 3 in [38]), the effects of keto-derivates on AmB-channel conductance cannot be attributed to the changes of the membrane dipole potential, but rather may proceed from the interaction of these two sterols with amphotericin B.

**Table 1:** Dependence of AmB single channels characteristics (conductance at zero transmembrane voltage, *G^0^*, and mean dwell time, *τ*) on sterol concentration in the membrane forming PC-solution.

Sterol concentration	Characteristic	6-ketocholestanol	7-ketocholestanol	5α-Androstan-3β-ol
33 mol%	*G^0^*, pS	7.0±0.5	8.0±1.0	11.5±1.0
	*τ*, ms	23±3	15±2	15±3
67 mol%	*G^0^*, pS	7.0±1.0	4.0±0.8	18.0±1.0
	*τ*, ms	38±5	37±5	37±3

The bilayers were bathed in 2.0 M KCl 5 mM Hepes pH 7.0.

The 5α-androstan-3β-ol is a fully saturated sterol without a hydrocarbon “tail”. It's effect on ϕ*_d_* is unknown. The absence of a hydrophobic tail may determine a localization of 5α-androstan-3β-ol molecules closer to the water-membrane interface, which leads to a more significant contribution of 5α-androstan-3β-ol dipoles to the ϕ*_d_* than other sterol molecules. As one can see from the data presented in Table 1, 5α-androstan-3β-ol increased the AmB-channel conductance in a concentration dependent manner: *G^0^*≈12 pS at 33 mol%, *G^0^*≈16 pS at 40 mol%, and *G^0^*≈18 pS at 67 mol%. Consequently, one can assume that 5α-androstan-3β-ol increases the membrane dipole potential. Indeed, we found that the addition of methyl-β-cyclodextrin up to 8.7 mM in the membrane bathing solution, which is known to remove sterol molecules from the membrane [38], led to an increase in K^+^-nonactin steady-state conductance of the bilayer containing 67 mol% 5α-androstan-3β-ol. This means that the decrease in 5α-androstan-3β-ol concentration in the membrane leads to a reduction of the bilayer dipole potential. It should be noticed that the specific interaction between this sterol and amphotericin may also contribute to the channel conductance as in the cases of 6-ketocholestanol and 7-ketocholestanol.

**Figure 4:**
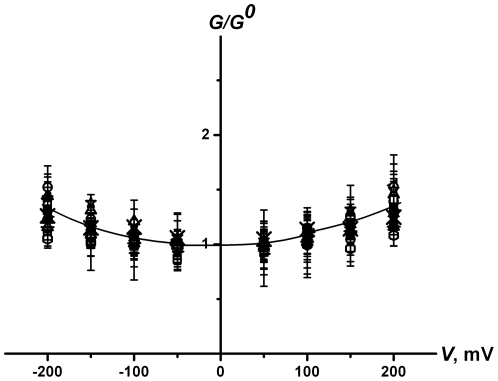
The voltage dependence of the ratio of AmB-channel conductance to conductance at zero transmembrane potential (*G*/*G^0^*). The membranes were bathed in 2.0 M KCl 5 mM Hepes pH 7.0. Bilayers were made from: (▪) – PC∶Chol (67∶33 mol%), (□) – PC∶Erg (67∶33 mol%), (•) – PC: 6-ketocholestanol (67∶33 mol%), (○) – PC: 7-ketocholestanol (67∶33 mol%), (×) – PC:5α-Androstan-3β-ol (67∶33 mol%). Solutions bathing membranes made from PC∶Chol (67∶33 mol%) contain: (▸) – 20 µM phloretin, (◂) – 20 µM quercetin, (▵) – 5 µM RH 421, (▴) – 5 µM RH 237, (◊) – 5 µM RH 160. Solutions bathing membranes made from PC∶Erg (67∶33 mol%) contain: (_*_) – 20 µM phloretin, (—□) – 20 µM quercetin, (+) – 5 µM RH 421, (—○) – 5 µM RH 237, (—▵) – 5 µM RH 160.

Figure 4 presents the voltage dependences of the ratios of AmB-channel conductance to *G^0^* for all investigated systems. It is seen that the shape of the conductance-voltage curves does not practically depend on a dipole modifier or sterol nature. The observed independence most likely means that the AmB-pore geometry is not influenced by ϕ*_d_* or interaction between a modifier and AmB. At the same time, one can reasonably think that the preferentially anionic character of the transport through AmB channels is also reserved for all the modifiers studied. If there were a significant change in the cation/anion selectivity the proper change in the conductance-voltage curves would be observed, as it took place in case of syringomycin E channels [41].

Along with the cases of Chol and Erg (Fig. 3) AmB-channel dwell time increased with an increasing concentration of 6-ketocholestanol, 7-ketocholestanol or 5α-androstan-3β-ol in the membrane forming solution (Table 1). Furthermore, in bilayers containing these sterols, the pore life time was smaller than in Chol-containing membranes. The data are in agreement with the findings of Smondyrev and Berkowitz [42] that the presence of the keto-group decreases membrane order and condensation due to a sterol shift towards the polar region closer to the interface. One can predict the same mechanism for 5α-androstan-3β-ol taking into account the absence of a hydrophobic tail in its molecule.

It has been shown that the interaction of certain drugs with cell membranes may depend on ϕ*_d_* [43]–[45]. Because the therapeutic and toxic effects of polyenes are due to their channel-forming activity in cell membranes, identifying opportunities for its regulation by the membrane dipole modifiers in the model systems (artificial planar bilayers) might be important for further inquiry on cell systems to achieve therapeutic effectiveness.
